# Role of BC040587 as a predictor of poor outcome in breast cancer

**DOI:** 10.1186/s12935-014-0123-7

**Published:** 2014-11-26

**Authors:** Yayun Chi, Sheng Huang, Lin Yuan, Mengying Liu, Naisi Huang, Shuling Zhou, Bingqing Zhou, Jiong Wu

**Affiliations:** Breast Cancer Institute, Fudan University Shanghai Cancer Center, Shanghai, 200032 China; Department of Breast Surgery, Fudan University Shanghai Cancer Center, Shanghai, 200032 China; Department of Pathology, Fudan University Shanghai Cancer Center, Shanghai, 200032 China; Department of Oncology, Shanghai Medical College, Fudan University, Shanghai, 200032 China

**Keywords:** BC040587, Breast cancer, Tumor suppressor, Prognosis, Long non-coding RNA

## Abstract

**Background:**

Accumulating studies have focused on the oncogenic and tumor suppressive roles of the newly identified lncRNAs. A novel lncRNA BC040587 in 3q13.31 locus which exists frequent copy number alterations was found to be associated with poor survival of osteosarcoma patients. However, its role in breast cancer (BC) remains unknown. The aim of this study was to examine the expression pattern of BC040587 in BC and to evaluate its biological role and clinical significance in prediction of prognosis.

**Methods:**

Expression of BC040587 was analyzed in 20 pairs of BC cancer tissues and adjacent noncancerous tissues (ANCT), also in 151 BC tissues, 9 BC cell lines and one normal breast cell line by quantitative reverse-transcription polymerase chain reaction (qRT-PCR). Differences between groups were tested for significance using Student’s t-test (two-tailed). Then we analyzed the potential relationship between BC040587 expression and clinic pathological features of BC patients. The correlation was analyzed by SPSS software.

**Results:**

It showed that BC040587 expression was down regulated both in BC samples and in BC cell lines compared with corresponding normal control. BC040587 expression was correlated with menopausal status (*p* = 0.040) and tumor differentiation (*p* = 0.035). The Kaplan-Meier survival curves indicated that the overall survival (OS) was significantly poor in low BC040587 expression BC patients (*p* = 0.023). Furthermore, expression of BC040587 was significantly associated with worse prognosis and was shown to be an independent prognostic marker breast cancer (*p* = 0.032). Our studies indicate that BC040587 may represent a new marker of prognosis in breast cancer.

**Conclusion:**

Our studies indicate that BC040587 is significantly down-regulated in BC tissues and BC cell lines. BC040587 may represent a new marker of prognosis in breast cancer.

## Introduction

Accumulating evidence show that Long non-coding RNAs (lncRNAs) play more and more important roles in a wide range of biological processes. Aberrant lncRNAs expression are involved in many cancers, such as glioma [1,2], lung [3,4], breast [5-7], and liver cancers [8-10]. LncRNA are also found to function as new regulators in cancer development [11]. Like protein-coding genes and miRNAs, lncRNAs can function as oncogenes or tumor suppressors during cancer progression [12]. For example, BANCR was found overexpressed in melanoma and required for full migratory capacity of melanoma cells by upregulating CXCL11, an important gene involved in cell migration [13]. lncRNA CRNDE which was highly elevated in many solid tumors and in acute myeloid leukemias (AML) can promote cell growth and suppress apoptosis, which supports a role for CRNDE as a mediator of oncogenesis [14,15]. LncRNA MEG3 is found markedly decreased in glioma tissues and ectopic expression of lncRNA MEG3 inhibited cell proliferation and promoted cell apoptosis via p53 activation in human glioma. Also, the expression of lncRNA MEG3 was associated with meningioma pathogenesis and progression [16,17].

Expression profiling of lncRNAs in human tumors has identified their association with diagnosis, staging, progression, prognosis, and response to treatment [12,18,19].

Many lncRNAs such as HOTAIR, which has been implicated in several cancers, such as lung cancer [4,20], gastric cancer [21], esophageal squamous cell carcinoma [22], colorectal cancers [23], have already shown their potential as novel independent biomarkers for early diagnosis and prognosis prediction in cancer. Meanwhile, lncRNA DD3 is also found to be a very sensitive and specific biomarker for the detection of prostate tumor cell [24,25]. Another recently reported study have shown that lncRNA HOTTIP and HOXA13, which expression were associated with metastasis and survival in HCC patients revealed their possible role of predictive biomarker of HCC [26].

Also more and more lncRNA are found to be associated with breast cancer outcomes. For example, expression of CCAT2, a novel long non-coding RNA in breast cancer was found to be related with clinical outcomes [5]. Jiang and his group also identified 122 putative breast cancer-associated long intergenic non-coding RNA loci by high density SNP array analysis [6]. Recent studies show that breast cancer patients with high HOTAIR expression had lower risks of relapse and mortality than those with low HOTAIR expression [27]. So seeking novel molecular lncRNA biomarkers of breast cancers is very important and helpful for its clinical diagnosis and management.

Previous studies identified 3q13.31 as a novel region of cooperatively acting tumor suppressor genes. BC040587 was located in the region 3q13.31, and it was found to be associated with poor survival of osteosarcoma patients [28]. Few studies were carried out in the other tumor type. The aim of this study was to examine the role of BC040587 in breast cancer and to confirm whether the expression of BC040587 was aberrant in breast cancer tissues and cancer cell lines and whether it was associated with poor prognosis in breast cancer.

## Results

### BC040587 show low expression in breast cancer tissues

**Figure 1:**
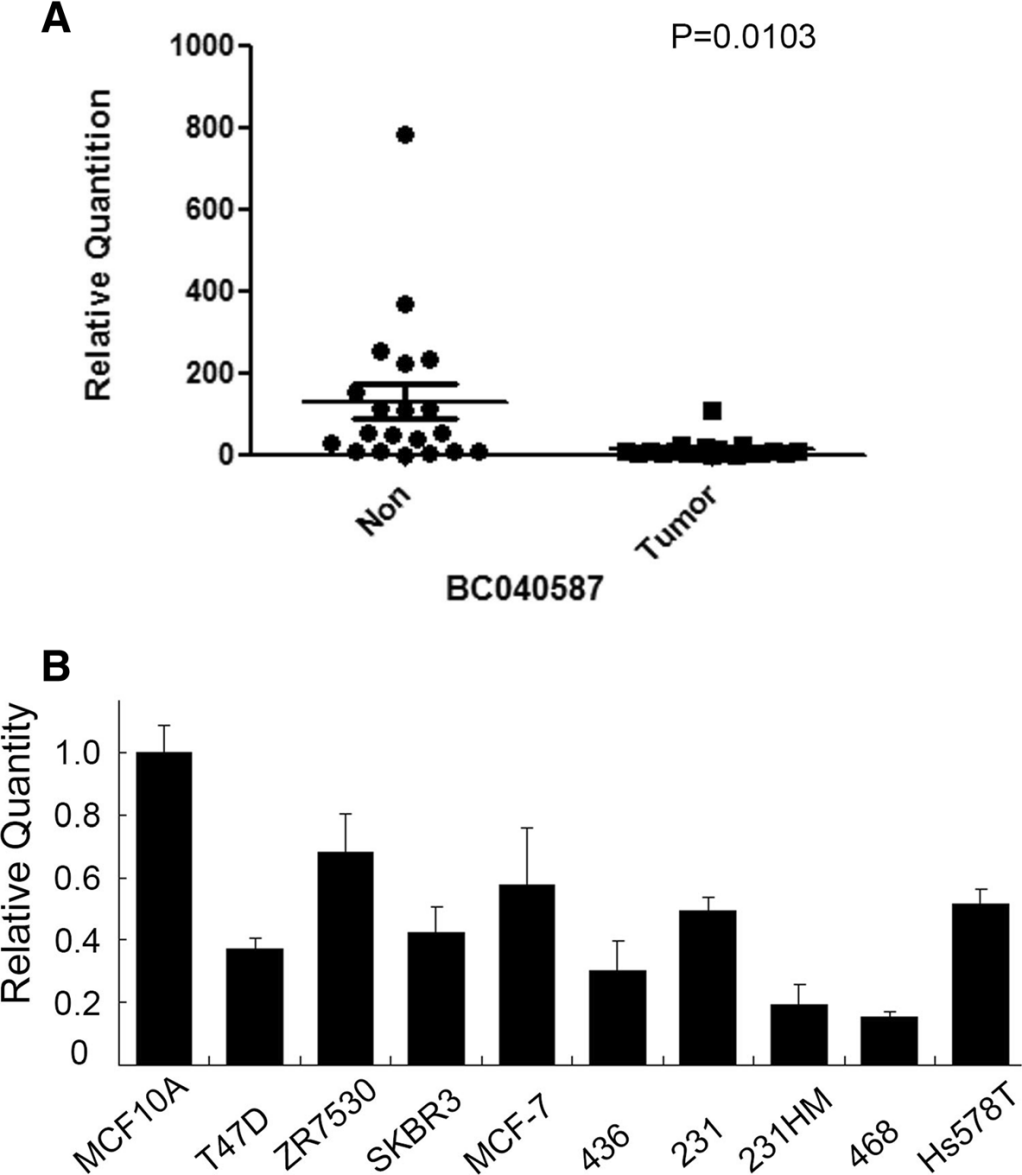
**BC040587 show low expression in breast cancer tissues and breast cancer cell lines. A**. Comparison of BC040587 expression levels between 20 pairs of BC tumor tissues and ANCT normal tissues by qRT-PCR analysis. GAPDH was used as an internal control (p < 0.01). **B**. Comparison of BC040587 expression levels between 9 BC cell lines and normal MCF10A cells by qRT-PCR analysis. GAPDH was used as an internal control. Abbreviations represent that 231, MDA-MB-231; 231HM, lung highly metastasis MDA-MB-231; 436, MDA-MB-436; 468, MDA-MB-468.

In order to examine the expression profile of BC040587 in breast cancer, we first detected the expression of BC040587 in 20 pairs of breast cancer tissues and it normal tissues. GAPDH was used as an internal control. 2^-ΔCt^ values were used to determine their relative expression. As shown in Figure 1A, BC040587 showed much lower expression in breast cancer tissues than in normal tissues. The *p* value was 0.01 and much less than 0.05. These data indicated that BC040587 might serve as a tumor suppressor in breast cancer.

### Expression profile of BC040587 in breast cancer cell lines and normal MCF10A cells

The expression of BC040587 was further examined in 9 breast cancer cell lines compared with the normal breast cell MCF10A. As shown in Figure 1B, compared with normal breast cancer cell line MCF10A, the breast cancer cells had much lower BC040587 expression. These data further suggested that expression of BC040587 was relatively low in breast cancer and BC040587 was a candidate tumor suppressor in breast carcinogenesis.

### Correlations between BC040587 expression and clinical characteristics

To identify the clinical relevance of BC040587 expression in breast cancer, correlation between BC040587 expression and clinic pathological parameters such as age, menopausal status, histological grade, tumor size, lymph node status and TNM stage were examined (Table 1). To assess the correlation of BC040587 expression with clinicopathologic data, the expression levels of BC040587 in tumor tissues were categorized as low or high in relation to the mean value. Of the 151 breast cancer patients, 76 cases were in BC040587 high group, and the other 75 cases were in the BC040587 low group. Chi-Square tests are used to determine the cutoff value of BC040587 high and BC040587 low group for the study. BC040587 expression in breast cancer was significantly correlated with menopausal status (*p* = 0.04), tumor differentiation (*p* = 0.035). Postmenopausal patients had a relatively low BC040587 expression level. Moreover, patients with lower BC040587 expression tended to have a higher risk of poor grade of tumor differentiation. However, BC040587 expression in breast cancer was not associated with other parameters such as age (*p* = 0.675), tumor location (*p* = 0.79), tumor size (*p* = 0.356), ER status (*p* = 0.828), PR status (*p* = 0.900), P53 staus (*p* = 0.217), Ki67 status (*p* = 0.844), lymph node status (*p* = 0.489) and TNM stage (*p* = 0.964) (Table 1).

**Table 1:** **Relationship between BC040587 expression and clinicopathological features in breast cancer patients**

	**loc285194**				
**Characteristics**	**Low**	**High**	**n**	**X** ^**2**^	***p***
Age(years)				0.175	0.675
<50	32 (47.8%)	35 (52.2%)	67		
≥50	43 (51.2%)	41 (48.8%)	84		
Menopausal status				4.199	**0.040**
Pre	28 (40.6%)	41 (59.4%)	69		
Post	47 (57.3%)	35 (42.7%)	82		
MT Family Hix				0.028	0.867
No	61 (48.8%)	64 (51.2%)	125		
Yes	14 (48.3%)	15 (51.7%)	29		
Tumor size (cm)				0.853	0.356
≦2 cm	22 (44%)	28 (56%)	50		
>2 cm	52 (52%)	48 (48%)	100		
Node status				0.479	0.489
Negative	36 (45%)	44 (55%)	80		
Positive	33 (50.8%)	32 (49.2%)	65		
ER status				0.047	0.828
Negative	26 (48.1%)	28 (51.9%)	54		
Positive	47 (50%)	47 (50%)	94		
PR status				0.016	0.900
Negative	28 (50%)	28 (50%)	56		
Positive	46 (48.9%)	48 (51.1%)	94		
HER-2 status				0.342	0.559
Negative	44 (51.8%)	41 (48.2%)	85		
Positive	31 (47%)	35 (53%)	66		
P53 status				1.525	0.217
Negative	25 (44.6%)	31 (55.4%)	56		
Positive	31 (56.4%)	24 (43.6%)	55		
Ki67 status				0.039	0.844
Negative	35 (50.7%)	34 (49.3%)	69		
Positive	20 (48.8%)	21 (51.2%)	41		
Differentiation				4.468	**0.035**
Well	34 (43%)	45 (57%)	79		
Poor	35 (61.4%)	22 (38.6%)	57		
TNM stage				0.002	0.964
III	53 (47.3%)	59 (52.7%)	112		
III	15 (46.9%)	17 (53.1%)	32		

*Abbreviations*: *ER* estrogen receptor, *HER-2* human epidermal growth factor receptor 2, *PR* progesterone receptor, *MT Family Hix* malignant tumor family history. Symbol Bold Data mean *p*<0.05.

### Association between BC040587 expression and patient survival

**Figure 2:**
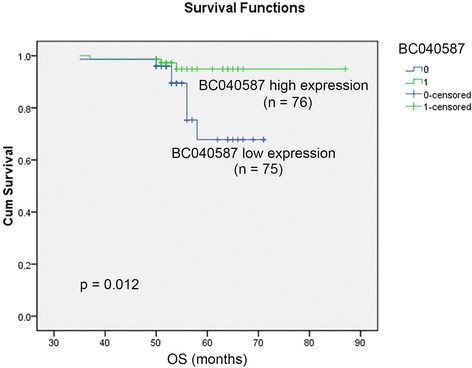
**Kaplan–Meier survival curves of patients with breast cancer based on BC040587 expression status.** Patients in the lower expression group had significantly poorer prognosis than those in low expression group (*p* < 0.05).

All patients were followed up for at least 5 years. Overall survival curves in low and high BC040587 expression groups were shown in Figure 2, and the overall survival was significantly lower in patients with lower BC040587 expression than those with high expression (*p* = 0.012). The Disease free survival showed no significance (Data not shown).

Univariate analysis (Log-rank test) of prognostic parameters for OS was performed. As shown in Table 2, patients with low BC040587 were significantly associated with a poorer OS (*p* = 0.031), HR positive patients were also associated with a good OS (*p* = 0.045). Multivariate analysis using Cox’s regression model was performed as shown in Table 3. HR status (*p* = 0.050) was shown to be border-line significant in multivariate analysis. And BC040587 expression (*p* = 0.032), and lymph node status (*p* = 0.047) were independent prognostic factors.

**Table 2:** **Univariate regression model of prognostic covariates in BC patients**

	**HR**	**95.0% CI**		***p*** **value**
**Variable**		**Lower**	**Upper**	
Age(<50/> = 50)	1.008	0.350	2.908	0.988
Menopause(positive/negative)	1.908	0.598	6.093	0.275
Differentiation(good/bad)	2.441	0.816	7.303	0.111
MT Family Hix(positive/negative)	1.276	0.355	4.584	0.708
Tumorsize(≦/>2 cm)	1.374	0.430	4.388	0.592
Lymph node(positive/negative)	3.125	0.828	11.792	0.093
TNM( I + II/III)	0.672	0.145	3.122	0.612
HR status (positive/negative)	0.326	0.109	0.974	**0.045**
HER2 status (positive/negative)	1.358	0.650	2.837	0.416
BC040587 (high/low)	0.245	0.068	0.879	**0.031**

*Abbreviations*: *CI* confidence interval, *HR status* hormone receptor status, *HER-2* human epidermal growth factor receptor 2, *HR* hazard ratio, *MT Family Hix* malignant tumor family history. Symbol Bold Data mean *p*<0.05.

**Table 3:** **Multivariate analysis of clinicopathological factors for overall survival in BC patients**

	**HR**	**95.0% CI**		***p*** **value**
**Variable**		**Lower**	**Upper**	
Lymph node(positive/negative)	3.891	1.014	14.907	**0.047**
TNM(I + II/III)	0.152	0.021	1.103	0.063
HR status (positive/negative)	0.335	0.112	1.000	**0.050**
HER2 status (positive/negative)	1.663	0.581	4.763	0.343
BC040587 (high/low)	0.248	0.069	0.890	**0.032**

*Abbreviations*: *CI* confidence interval, *HR status* hormone receptor status, *HER-2* human epidermal growth factor receptor 2, *HR* hazard ratio. Symbol Bold Data mean *p≦*0.05.

## Discussion

Breast cancer is the most common female cancer and among the most frequent causes of cancer mortality in women worldwide. Also, cancer is a complex disease, involving various changes in gene expression. As a newly discovered class of non-coding genes, altered expression of lncRNAs is frequently observed in human cancers, including breast cancer [1]. The alterations in the expression, the primary structure, secondary structure as well as their binding proteins are often associated with metastasis, invasion and patient survival. It suggested that the potential role of lncRNAs should be further investigated.

We present evidence here that BC040587 was down regulated in the breast cancer tissues as well as breast cancer cells and it low expression may contribute to the patient poor survival.

BC040587 is located at chromosome 3q13.31 [2]. As the 3q13.31 locus harbors frequent focal copy number alterations (CNAs) and loss of heterozygosity in primary osteosarcoma samples [3], it implies that BC040587 may function as a potential tumor suppressor in osteosarcoma samples [4,2]. However, little is known about the role of BC040587 in breast cancer.

We show that expression of BC040587 was down regulated in breast cancer tissues compared with the ANCT in 20 pairs of patients (*p* < 0.05). Furthermore, expression of BC040587 was attenuated in most breast cancer cell lines compared with the normal breast cancer cell line MCF10A. These data suggest that BC040587 may also function as a tumor suppressor in breast cancer. Then relationship between BC040587 expression and clinicopathological features in breast cancer patients was then examined. It showed that expression of BC040587 was relatively lower in the postmenopausal stage than in the premenopausal stage (*p* = 0.040). It was supposed that the expression of BC040587 might be effected by the endocrine system. There are no correlated reports. However, expression of BC040587 had no significant association with ER and PR status in our research. It needs further investigation. Furthermore, it showed that low expression of BC040587 was related to poor tumor differentiation (*p* = 0.035). These data suggest that BC040587 may function as a potential tumor suppressor and a predictive biomarker in breast cancer.

The overall survival was significantly lower in patients with lower BC040587 expression than those with high expression (*p* = 0.012) which was consistent with reports of tumor-suppressive role of BC040587 in osteosarcoma. Univariate analysis (Log-rank test) of prognostic parameters for OS showed that patients with low BC040587 were significantly associated with a poorer OS (*p* = 0.031). HR positive patients were also significantly associated with a good OS (*p* = 0.045). Multivariate analysis was examined to found BC040587 expression (*p* = 0.032) was an independent prognostic indicator for OS in addition with lymphatic metastasis (*p* = 0.047) and HR status (*p* = 0.05) (Table 3).

As we known, lncRNAs impact cellular functions through various mechanisms, such as interactions with chromatin remodeling proteins [29,30], they are able to regulate other non-coding RNAs, in particular microRNAs. For example, loc285194 is a p53-regulated tumor suppressor, which acts in part through repression of miR-211 [31]. We speculated that BC040587 might regulated by other powerful genes or it could regulate other genes to play its special roles and function as a tumor suppressor in breast cancer. However, little was known about the BC 040587 signaling pathway, it needs further investigation.

## Conclusion

BC040587 was significantly down-regulated in BC compared with ANCT. Expression of BC040587 was low in the postmenopausal patients with poor tumor differentiation and poor survival. Our results suggest that attenuated expression of BC040587 may play an important role in BC and it could be an independent prognostic biomarker in breast cancer.

## Materials and methods

### Patients’ samples

A total of 151 primary breast cancer samples of stage I to III invasive ductal carcinoma cases and ANCT were collected randomly at the Department of Breast Surgery in Fudan University Shanghai Cancer Center (FDUSCC, Shanghai, P.R. China). Each case was given a unique identifier and linked to a database containing clinical-pathological data. ANCT means the normal breast tissue and it was diagnosed by the pathologists through H.E. staining. The tumors were assessed according to the WHO classification by two academic pathologists. In addition, the pathological data including (ER, PR), HER2, P53 and Ki67 status/expression were assessed and diagnosed by the pathologists based on the ASCO breast cancer guideline. Patient information and tumor pathology are summarized. This study was approved by the Ethical Committee of Fudan University Shanghai Cancer Center for Clinical Research. The written informed consents were obtained from all the patients.

### Cell culture and regents

Fifteen breast cell lines were obtained from cell bank of our lab. ZR-75-30, MCF-7, SKBR-3 and T47D cells were grown using 1640 medium. MDA-MB231, MDA-MB231HM cells were cultured using F15. MDA-MB436, MDA-MB468, Hs578T cells were cultured with DMEM medium. MCF10A were cultured with F12/DMEM 1:1 medium. All medium are with 10% FBS, 100 units/ml penicillin, and 100 ug/ml streptomycin. The cells were cultured at 37°C and 5% CO_2_.

### RNA extraction and Quantitative RT-PCR

Total RNA was extracted using TRIzol reagent (Invitrogen). After converting total RNA to cDNA in a reverse transcription (RT) reaction, qPCR were used to quantitate the mRNA expression levels. To detect BC040587 expression, we used the SYBR.

Green method with primers listed below: *BC040587* exon 2 forward 5′ TAACAAGATTCACCTGCCAACC 3′ and *BC040587* exon 2 reverse 5′ TGAGATCCAGAGTGTGCTGAAA 3′. GAPDH was used as an internal control. 2^-delta Ct^ values were used to determine their relative expression.

### Statistical analysis

Analyses were performed using SPSS software. Kaplan-Meier survival analysis was also performed using SPSS. Differences with *p*-values <0.05 are considered significant. Univariate analysis were used in multivariate analysis on the basis of Cox proportional hazards model. Two-sided p-values were calculated and a probability level of 0.05 was chosen for statistical significance.
